# Prior Storm Experience Moderates Water Surge Perception and Risk

**DOI:** 10.1371/journal.pone.0062477

**Published:** 2013-05-30

**Authors:** Gregory D. Webster, Duzgun Agdas, Forrest J. Masters, Corey L. Cook, Amanda N. Gesselman

**Affiliations:** 1 Department of Psychology, College of Liberal Arts and Sciences, University of Florida, Gainesville, Florida, United States of America; 2 Engineering School of Sustainable Infrastructure & Environment, College of Engineering, University of Florida, Gainesville, Florida, United States of America; 3 Psychology Department, Skidmore College, Saratoga Springs, New York, United States of America; CSIC-Univ Miguel Hernandez, Spain

## Abstract

**Background:**

How accurately do people perceive extreme water speeds and how does their perception affect perceived risk? Prior research has focused on the characteristics of moving water that can reduce human stability or balance. The current research presents the first experiment on people's perceptions of risk and moving water at different speeds and depths.

**Methods:**

Using a randomized within-person 2 (water depth: 0.45, 0.90 m) ×3 (water speed: 0.4, 0.8, 1.2 m/s) experiment, we immersed 76 people in moving water and asked them to estimate water speed and the risk they felt.

**Results:**

Multilevel modeling showed that people increasingly overestimated water speeds as actual water speeds increased or as water depth increased. Water speed perceptions mediated the direct positive relationship between actual water speeds and perceptions of risk; the faster the moving water, the greater the perceived risk. Participants' prior experience with rip currents and tropical cyclones moderated the strength of the actual–perceived water speed relationship; consequently, mediation was stronger for people who had experienced no rip currents or fewer storms.

**Conclusions:**

These findings provide a clearer understanding of water speed and risk perception, which may help communicate the risks associated with anticipated floods and tropical cyclones.

## Introduction

Moving water can be a deadly force. In the U.S. alone, nearly 100 people die in rain-related floods every year – many while attempting to cross shallow but swiftly moving water [1]. Storm surge – the ocean flooding that accompanies landfalling hurricanes – can be catastrophic, claiming nearly 600 of about 1,500 lives lost in and around New Orleans in 2005 [2] and nearly a quarter million lives in Bangladesh in 1970 [3]. Although advanced warnings are given for storm surges and most flash floods, many residents “ride out” storm surges or attempt to cross flooded streams or roadways when they should not [4]. One reason for people's risky decisions may be their misperceptions of moving water, the force it can generate, and its associated dangers. The current research presents the first experimental study of people's perception of moving water at different speeds and depths in conjunction with their assessments of personal risk.

### Prior Research

Prior flooding research has taken two approaches. The first has primarily focused on flood characteristics and community demographics to obtain projected estimates of damage and fatalities. This line of research has advanced a framework for predicting flood-related injuries and deaths [5], and has suggested that risk-taking (e.g. driving across flooded roadways) can be affected by flood characteristics (e.g., depth, speed), and efficiency of emergency response systems [6]. Collectively, this research aims to understand large-scale factors that affect flood disasters rather than person-level contributors to risk-taking.

The second approach focuses on the characteristics of moving water that can negate human stability or balance. Human stability in moving water is largely a function of water depth and speed. In small-scale experiments (*N*s * = *7–20), participants were immersed in laboratory flumes and exposed to differing water speeds and depths. Researchers determined conditions causing instability, and derived formulas to determine the threshold for imbalance in flowing water [7], [8]. This was extended to a one-person field experiment in which researchers built structures (sluice gates) to control downstream water depth and speed in a river channel [9]. While informative, this research is limited by an overreliance on balance thresholds and small samples [10], and has not included perceptual factors.

### The Present Research

**Figure 1:**
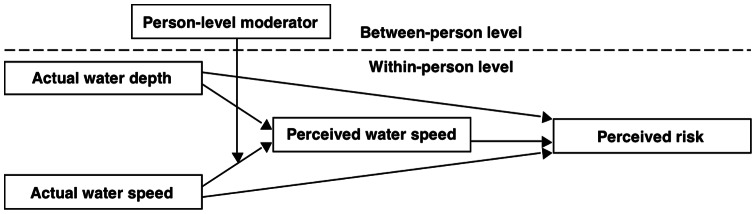
Multilevel moderated mediation model. Perceived water speed (partially) mediates the direct relationships between (a) actual water speed perception and risk (b) water depth and risk (level 1). The first relationship is moderated by prior storm and rip current experience (level 2).

How do people perceive fast-moving water? How accurate are their perceptions? What makes some people more accurate than others? To answer these questions, we designed a within-person experiment that immersed people in various water depths and speeds, and asked them to estimate the speed of the moving water and how much personal risk they felt. Before the experiment, we asked them about prior experience with rip current and tropical cyclones (i.e., tropical storms with sustained winds ≥39 mph [63 km/h or 17.5 m/s]; hereafter referred to as “storms”). We made two predictions. First, we expected that the relationship between actual and perceived water speed would be more accurate (less overestimating) among people with more prior experience. Second, we expected that perception of water speed would at least partially mediate the direct relationship between actual water speed and risk, and that this direct relationship would be stronger among people with more storm experience (moderated mediation; Figure 1).

## Methods

### Ethics Statement

Ethical standards outlined by the American Psychological Association (APA) were followed in the conduct of this research, which was approved by the University of Florida Institutional Review Board. All participants gave their signed consent prior to partaking in the experiment. Following APA guidelines, data can be requested for five years post publication.

### Participants and Procedure

Seventy-six University of Florida students (18 women, 58 men) aged 18–40 years (*M* = 23.47, *SD*  = 4.68) participated in the study. Participants were surveyed on their prior experiences with extreme weather (prior storm and rip current experience). Next, participants donned protective gear (waders and raincoats) and a harness that attached to a metal cage that could be lowered into the water flume at various depths. Participants were then immersed in three moving water speeds (0.4, 0.8, 1.2 m/s) at two different depths (0.45, 0.90 m) resulting in a 2×3 within-person experiment. Between each immersion event (which lasted ≈20 s), participants communicated their water speed estimate (in mph or m/s) and their risk estimate of personal injury on a scale of 0 (*no perceived risk*) to 10 (*dangerous*). The testing conditions (gear, exposure time, water speed and depth) were identical across participants; the order of water speeds and depths were randomized. To prevent bias, participants were given no information on the actual water speeds until after the experiment. The same 76 people also participated in a separate wind perception experiment [11]; however, the only data overlap between [11] and the present experiment was demographic information (e.g., number of storms experienced).

### Moving Water Flume

We designed a rectangular (70×4×4 ft [21.34×1.22×1.22 m]) flume (open water channel) for the experiment. Participants were harnessed to a steel cage that was lowered into the flume using a hydraulic winch. An operator controlled the hydraulic lifting system and the flume's water speed. An onboard jet system from a personal watercraft (Sea-Doo) accelerated the water through the flume. The water speed was initially calibrated using force measurements on drag plates immersed in the flow to ensure consistency between tests. Reported values refer to the surface speed of the water, which was later determined by quantifying the amount of time it took for a floating object to travel across a set distance near the test section. Turbulence characteristics were not measured.

### Data Analysis

Because repeated estimates were nested within participants, we analyzed the data with multilevel modeling (MLM) using HLM [12] and Mplus [13]. Using a maximum likelihood algorithm, MLM estimates within- and between-person effects simultaneously. We modeled within-person variance in water speed and risk perception at level 1 and between-person variance at level 2 as a function of means (intercepts) and, in some models, individual differences in rip current experience (−0.5 =  no, 0.5 =  yes) or number of storms experienced (grand-mean-centered at 5.0 storms [*SD*  = 3.0]; i.e., the tropical storms with sustained winds ≥39 mph (63 km/h or 17.5 m/s), hereafter referred to in shorthand as “storms”). We also examined multilevel moderated mediation models [11], [14]–[16], where we expanded the MLM framework to include level-1 mediation with a dichotomous (rip current) or continuous (storms experienced) level-2 moderator (see [11] for examples).

## Results

### Descriptive Statistics

See Table 1 for descriptive statistics of perceptions of water speed and risk.

**Table 1:** Descriptive statistics for water speed and risk perceptions by actual water speed (m/s).

	Water speed perceptions (m/s)				Risk perceptions				
Actual water speed	Range	*Mdn*	Mean	*SD*	Range	*Mdn*	Mean	*SD*	*r*
All water speeds	0.00–33.53	2.27	3.81	4.14	0–10	3.0	3.39	2.45	.51*
At 0.45 m depth	0.00–22.35	2.27	3.50	3.47	0–9	3.0	2.75	2.01	.52*
0.4 m/s	0.00–8.94	0.90	1.19	1.31	0–4	1.0	1.07	1.04	.26*
0.8 m/s	0.33–15.00	3.00	4.04	3.20	0–7	3.0	3.08	1.70	.31*
1.2 m/s	0.50–22.35	4.49	5.26	3.94	0–9	4.0	4.12	1.85	.32*
At 0.90 m depth	0.15–33.53	2.29	4.12	4.71	0–10	4.0	4.02	2.68	.50*
0.4 m/s	0.15–7.60	0.72	1.23	1.29	0–6	1.0	1.20	1.22	.16
0.8 m/s	0.33–26.82	3.64	4.78	4.77	0–10	5.0	4.82	2.04	.26*
1.2 m/s	0.50–33.53	5.18	6.36	5.37	2–10	6.0	6.05	1.81	.31*

*Note.* Nesting *not* taken into account; data averaged across persons rather than examining data within persons. *r = * correlation between water perceptions and risk perceptions. *N*s  = 76 participants, 456 observations.

* *p*<.05.

### Water Speed Estimates as a Function of Actual Water Speed

**Figure 2:**
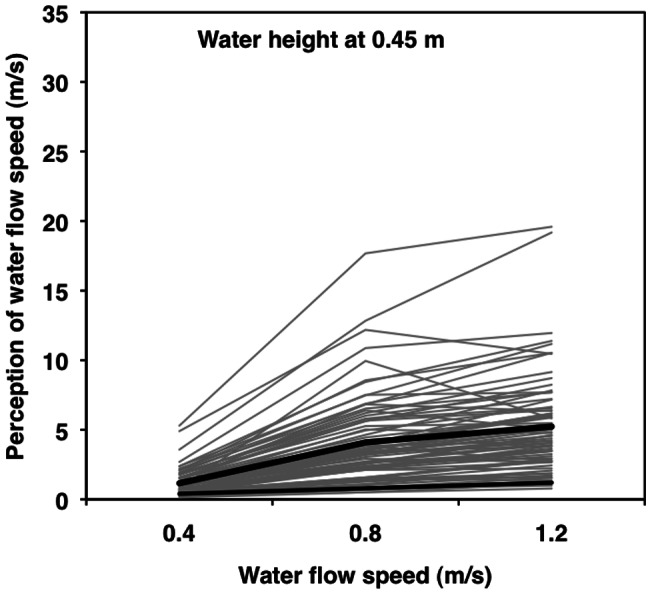
Spaghetti plot: Water speed: 0.45 m. Multilevel modeling results for perceived water speed as a function of water depth at 0.45 m and actual water speed (0.4 vs. 0.8 vs. 1.2 m/s). Thin grey lines represent individual predicted scores for 76 participants. The thick black line represents the average person. The thin black line represents a one-to-one relationship.

**Figure 3:**
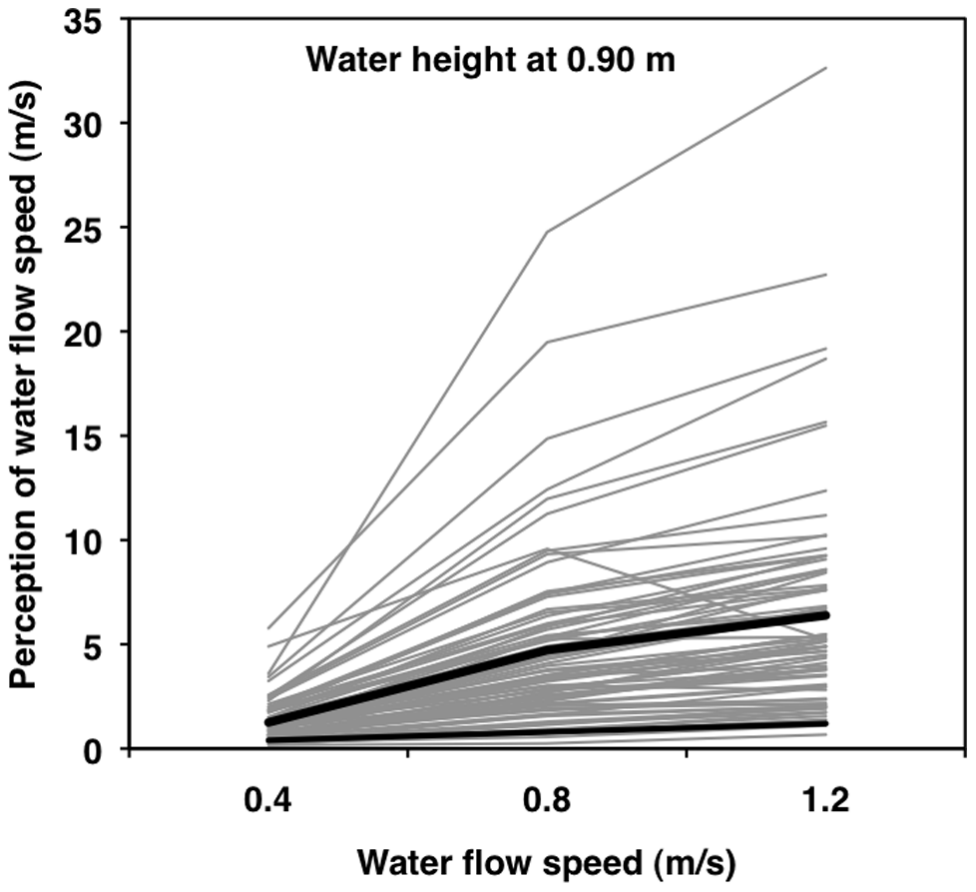
Spaghetti plot: Water speed: 0.90 m. Multilevel modeling results for perceived water speed as a function of water depth at 0.90 m and actual water speed (0.4 vs. 0.8 vs. 1.2 m/s). Thin grey lines represent individual predicted scores for 76 participants. The thick black line represents the average person. The thin black line represents a one-to-one relationship.

We tested models in two steps [17], starting with the “main effects” of actual water speed and depth at Step 1 and adding two 2-way interactions (speed^2^ and speed × depth) at Step 2 (Table 2). In Step 1, both actual water speed and water depth uniquely and significantly contributed to the average person's perception of water speed (Table 2, left, top). In Step 2, both speed^2^ and the speed × depth interaction were significant predictors of water speed perception (Table 2, left, bottom; Figures 2 and 3).

**Table 2:** Water speed perception and risk perception as functions of water depth (m) and the linear and quadratic effects of actual water speed (m/s).

Model or variable	Water speed perception			Risk perception		
	*b*	*t*_75_	*r*_p_^a^	*b*	*t*_75_	*r*_p_^a^
Model 1						
Intercept	3.81	11.60*	–	3.39	23.61*	–
Depth	1.39	2.36*	.26	2.82	10.64*	.78
Speed	5.75	11.16*	.79	4.94	27.08*	.95
Model 2						
Intercept	4.41	10.52*	–	3.95	20.74*	–
Depth	1.39	2.36*	.26	2.82	10.64*	.78
Speed	5.75	11.16*	.79	4.94	27.08*	.95
Speed^2^	−5.65	−5.02*	−.50	−5.24	−6.81*	−.62
Depth × speed	2.94	2.37*	.26	5.01	8.34*	.69

*Note.* ^a^Partial correlations (*r*_p_). *b  = * Unstandardized regression coefficient.

* *p*<.05.

**Figure 4:**
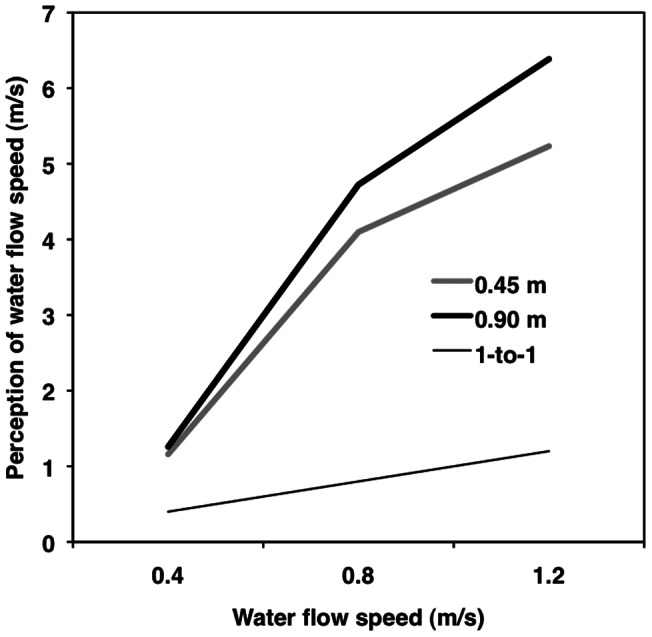
Perception of water speed by actual water speed and depth. Multilevel modeling results for perceived water speed as a function of water depth (0.45 [Grey] vs. 0.90 [Black] m) and actual water speed (0.4 vs. 0.8 vs. 1.2 m/s). The thin black line represents a one-to-one relationship.

Simple effects tests [17] showed that average perceived water speeds were significantly greater than actual water speeds for all actual water speeds and depths (Table 3, left; Figure 4). The simple slope between perceived and actual water speeds was computed for all six conditions of the 2×3 design to test the departure of people's perceptions from a one-to-one accuracy slope. At actual water speeds of 0.4 and 0.8 m/s, the simple slopes were significantly more positive than the one-to-one relationship at both water depths, suggesting that people became less accurate about the water speed function (departed from linearity) as water speeds increased (Table 3, right). At 1.2 m/s, however, the average participant's simple slope did not differ significantly from a one-to-one relationship regardless of water depth; people's perceptions of the water speed slope or function – but not the level – were fairly accurate.

**Table 3:** Simple effects: Water speed perception as a function of actual water speed.

	Intercept (difference from actual)			Slope (difference from 1-to-1)		
Actual water speed (m/s)	*b*	*t*_75_	*d*	*b*	*t*_75_	*d*
At 0.45 m						
0.4 m/s	0.76	5.53*	1.28	8.61	7.94*	1.83
0.8 m/s	3.30	8.76*	2.02	4.09	9.10*	2.10
1.2 m/s	4.03	9.17*	2.12	−0.42	−0.46	−0.11
At 0.90 m						
0.4 m/s	0.86	5.30*	1.22	9.27	7.61*	1.76
0.8 m/s	3.92	7.92*	1.83	4.75	9.22*	2.13
1.2 m/s	5.19	8.30*	1.92	0.23	0.29	0.07

*Note. N*s  = 76 participants, 454 observations (2 data points missing due to procedural error).

* *p*<.05.

#### Rip current

**Figure 5:**
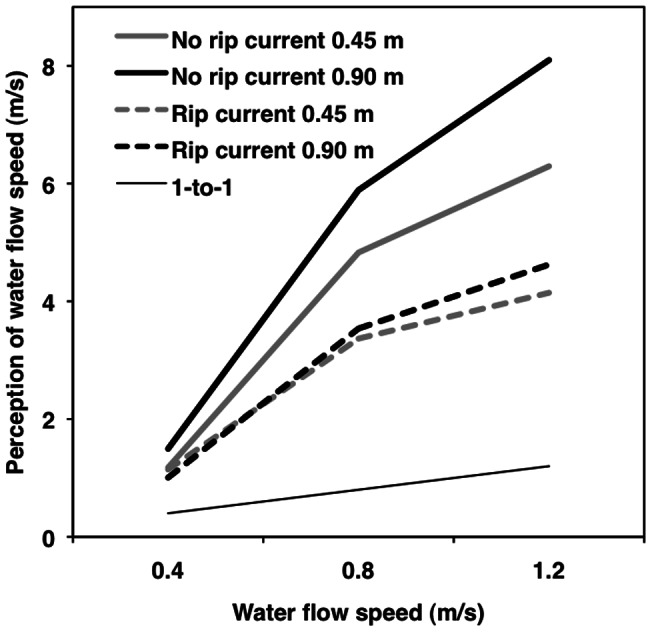
Moderation by rip current experience. Multilevel modeling results for perceived water speed as a function of water depth (0.45 [Grey] vs. 0.90 [Black] m), actual water speed (0.4 vs. 0.8 vs. 1.2 m/s), and prior rip current experience (yes vs. no). The thin black line represents a one-to-one relationship.

Prior experience with rip currents (yes vs. no) moderated only the linear effect of actual water speed on water speed perception (*b* = −3.18, *t*_73_  = −3.35, *p* = .001, *r*_p_  = −.37; Figure 5). Simple effects tests showed that people with no prior rip current experience had more positive (less accurate) slopes (*b* = 7.32, *t*_73_  = 8.70, *r*_p_  = .71) than people that had prior experience with rip currents (*b* = 4.14, *t*_73_  = −9.39, *r*_p_  = .74; *p*s <.001).

#### Number of storms

**Figure 6:**
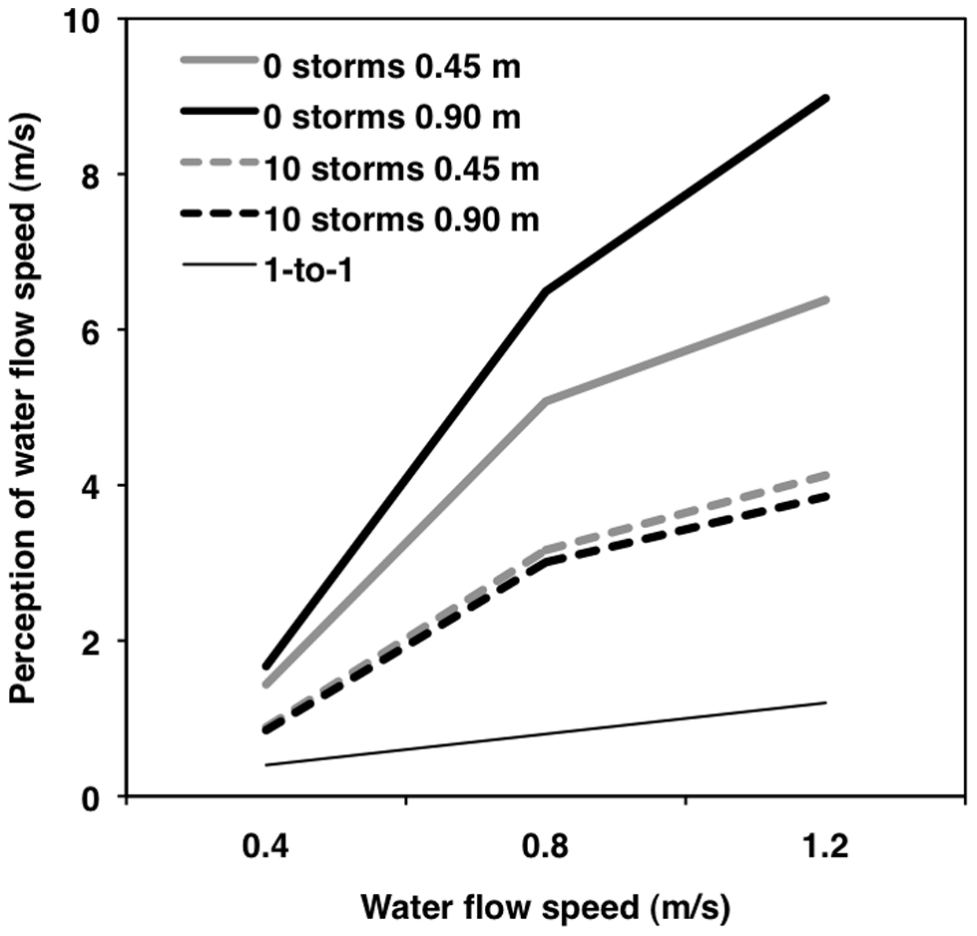
Moderation by storms experienced. Multilevel modeling results for perceived water speed as a function of water depth (0.45 [Grey] vs. 0.90 [Black] m), actual water speed (0.4 vs. 0.8 vs. 1.2 m/s), and number of storms experienced (0 vs. 10 or more). The thin black line represents a one-to-one relationship.

Number of storms experienced moderated only the linear effect of actual water speed on water speed perception (*b* = −0.38, *t*_73_  = −2.14, *p* = .036, *r*_p_  = −.24; Figure 6). Simple effects tests showed that people who had experienced no storms had more positive (less accurate) slopes (*b* = 7.65, *t*_73_  = 6.30, *r*_p_  = .59) than people who had experienced 10 or more storms (*b* = 3.90, *t*_73_  = 5.19, *r*_p_  = .52; *p*s <.001). It is unlikely that four participants experienced 10 or more tropical cyclones based on their age and historic data. The possible inaccuracies might relate to misperceptions about the environmental conditions that constitute tropical cyclones. Nevertheless, when we re-ran the model without these four participants, number of storms experienced still moderated the linear effect of actual water speed on perception of water speed (*b* = −0.34, *t*_69_  = −1.82, *p = *.072, *r*_p_  = −.21), although it was reduced to marginal significance.

### Risk as a Function of Actual Water Speed

We tested models in two steps (Table 2). In Step 1, both actual water speed and water depth uniquely and significantly contributed to the average person's risk perception (Table 2, right, top). In Step 2, both speed^2^ and the speed × depth interaction were significant predictors of risk perception (Table 2, right, bottom). None of these effects was significantly moderated by prior experience with storms or rip currents.

### Risk as a Function of Perceived Water Speed

We tested models in two steps (Table 4). In Step 1, both perceived water speed and water depth uniquely and significantly contributed to the average person's risk perception (Table 4, top). In Step 2, both perceived speed^2^ and the perceived speed × depth interaction were significant predictors of risk perception (Table 4, bottom).

**Table 4:** Risk perception as functions of water depth (m) and the linear and quadratic effects of perceived water speed (m/s).

Model or variable	*b*	*t*_75_	*r*_p_^a^
Model 1			
Intercept	3.39	23.61*	–
Depth	2.10	8.26*	.69
Perceived speed	0.89	14.97*	.87
Model 2			
Intercept	3.56	21.40*	–
Depth	2.24	9.09*	.72
Perceived speed	0.96	14.66*	.86
Perceived Speed^2^	−0.059	−7.51*	−.66
Depth × perceived speed	0.83	9.59*	.74

*Note.* ^a^Partial correlations (*r*_p_). *b  = * Unstandardized regression coefficient.

* *p*<.05.

Number of storms experienced moderated the linear (*b* = 0.041, *t*_73_  = 1.94, *p* = .056, *r*_p_  = .22) and quadratic (*b* = −0.0061, *t*_73_  = −2.88, *p* = .006, *r*_p_  = −.32) effects of perceived water speed on risk perception.^2^ Simple effects tests for people who experienced 10 or more storms showed significant linear (*b* = 1.14, *t*_73_  = 9.46, *r*_p_  = .74) and quadratic (b = −0.083, *t*_73_  = −6.39, *r*_p_  = .60) effects of perceived water speed on risk perception (*p*s <.001). Simple effects tests for people who experienced no storms showed weaker but still significant linear (*b* = 0.74, *t*_73_  = 6.06, *p*<.001, *r*_p_  = .58) – and marginal quadratic (*b* = −0.022, *t*_73_  = −1.87, *p* = .065, *r*_p_  = −.21) – effects of perceived water speed on risk perception. In sum, people who had experienced more storms had slopes that were more linear than those who had experienced fewer storms, and the extent to which they decelerated at greater perceived water speeds was also greater for those who had experienced more storms. When we re-ran this model without the four participants who reported experiencing 10 or more storms, number of storms experienced no longer marginally moderated the linear effect (*b* = 0.034, *t*_69  = _1.49, *p = *.14, *r*_p_  = .18) – but still moderated the quadratic effect (*b* = −0.0066, *t*_69  = _−2.89, *p = *.006, *r*_p_  = −.33) – of water speed perception on risk.

### Multilevel Moderated Mediation Models

Figure 1 shows a multilevel mediation model in which a between-person (level-2) moderator (prior experience with storms or rip currents) moderates the strength of the actual–perceived water speed relationship, which in turn alters the strength of the indirect effect on perceived risk (moderated mediation). In moderated mediation, the strength of a mediational relationship among three variables (e.g., *X* → *Med.* → *Y*) varies as a function of a fourth variable – a moderator (e.g., *Mod*.). For example, the relationship between sunny (vs. cloudy) days and frequency of “brain freezes” or “ice-cream headaches” (technically *sphenopalatine ganglioneuralgia*) is likely mediated by – or explained by – ice cream consumption. That this mediation is likely stronger in the summer than in the winter suggests that its strength is moderated by seasonal effects. Specifically, it could be that seasonality affects the strength of the relationship between sunny (vs. cloudy) days and ice cream consumption; moderation of this path alone is likely sufficient for moderated mediation to occur. In the context of the present study, we examined the extent to which the relationships between actual water speed and people's perceptions of persona risk were mediated by their perception of water speed, and whether variability in the strength of that mediation across people was explained by individual differences in prior experience with storms and rip currents.

In the moderated mediation results shown in Tables 5 and 6, we start by testing a baseline model predicting risk from depth and speed in the top half of the upper third of each table. This shows the direct, unmediated effects of actual water depth and speed on risk, controlling for the moderator (rip currents in Table 5, storms in Table 6). We then add the mediator to the model – people's estimates or perceptions of water speed – in the bottom half of the upper third of each table. These numbers describe the full mediation model (controlling for the moderator), where actual water depth and speed are predicting both people's estimates (or perceptions) of water speed and their risk, and people's estimates (or perceptions) of water speed are in turn predicting risk. In the middle and lower thirds of each table, we show the results of simple effects tests, showing differences in the strength of the mediation and different levels of the moderator. Specifically, the middle and lower thirds of Table 5 show the simple mediation patterns for people lacking and having prior rip current experiences (respectively), whereas the middle and lower thirds of Table 6 show the simple mediation patterns for people who have experienced zero and ten storms (respectively).

**Table 5:** Multilevel moderated mediation model.

Model or variable	Estimate (perception)				Risk			
	*b*	95% CI	*t*	*r*_p_^a^	*b*	95% CI	*t*	*r*_p_^a^
Controlling for rip currents								
Baseline								
Depth					2.79	[2.27, 3.31]	10.54*	.78
Speed					4.91	[4.56, 5.27]	27.09*	.95
Mediation								
Depth	1.37	[0.26, 2.49]	2.41*	.27	2.37	[1.91, 2.82]	10.16*	.77
Speed	5.73	[4.80, 6.66]	12.05*	.82	2.94	[2.30, 3.59]	8.94*	.72
Estimate					0.40	[0.26, 0.54]	5.50*	.54
Simple effects for people with no rip current experience								
Baseline								
Depth					2.68	[1.90, 3.46]	6.76*	.62
Speed					5.21	[4.73, 5.69]	21.22*	.93
Mediation								
Depth	2.36	[0.28, 4.44]	2.22*	.25	2.12	[1.41, 2.83]	7.53*	.66
Speed	7.32	[5.67, 8.97]	8.69*	.71	3.11	[2.30, 3.92]	2.22*	.25
Estimate					0.34	[0.20, 0.49]	4.60*	.47
Simple effects for people with rip current experience								
Baseline								
Depth					2.90	[2.21, 3.59]	8.27*	.70
Speed					4.62	[4.10, 5.14]	17.30*	.90
Mediation								
Depth	0.38	[−0.42, 1.19]	0.93	.11	2.62	[2.03, 3.20]	8.80*	.72
Speed	4.14	[3.27, 5.00]	9.39*	.74	2.78	[1.89, 3.67]	6.12*	.58
Estimate					0.46	[0.25, 0.66]	4.32*	.45

Water speed perception as a mediator of the relationship between water depth and actual speed predicting perceptions of risk, moderated by prior experience with rip currents.

*Note. N = *75 participants. ^a^Partial correlations (*r*_p_).

* *p*<.05.

**Table 6:** Multilevel moderated mediation model.

Model or variable	Estimate (perception)				Risk			
	*b*	95% CI	*t*	*r*_p_^a^	*b*	95% CI	*t*	*r*_p_^a^
Controlling for number of tropical cyclones experienced								
Baseline								
Depth					2.78	[2.26, 3.30]	10.51*	.78
Speed					4.92	[4.56, 5.28]	26.91*	.95
Mediation								
Depth	1.40	[0.26, 2.53]	2.42*	.27	2.36	[1.90, 2.82]	10.06*	.76
Speed	5.78	[4.80, 6.76]	11.55*	.80	2.95	[2.30, 3.59]	8.96*	.72
Estimate					0.40	[0.26, 0.53]	5.62*	.55
Simple effects for people who experienced zero tropical cyclones								
Baseline								
Depth					3.01	[1.82, 4.20]	4.94*	.50
Speed					5.18	[4.43, 5.93]	13.57*	.85
Mediation								
Depth	3.14	[−0.41, 6.70]	1.73†	.20	2.22	[1.20, 3.25]	4.24*	.44
Speed	7.66	[5.27, 10.04]	6.29*	.59	3.06	[1.93, 4.19]	5.30*	.53
Estimate					0.29	[0.07, 0.51]	2.57*	.29
Simple effects for people who experienced 10 or more tropical cyclones								
Baseline								
Depth					2.54	[1.64, 3.45]	5.50*	.54
Speed					4.67	[4.01, 5.34]	13.84*	.85
Mediation								
Depth	−0.35	[−2.38, 1.69]	−0.33	−.04	2.49	[1.66, 3.33]	5.86*	.57
Speed	3.90	[2.43, 5.38]	5.19*	.52	2.84	[2.01, 3.68]	6.70*	.62
Estimate					0.50	[0.32, 0.68]	5.42*	.54

Water speed perception as a mediator of the relationship between water depth and actual speed predicting perceptions of risk, moderated by number of tropical cyclones experienced.

*Note. N = *75 participants. ^a^Partial correlations (*r*_p_).

† *p*<.10. *p<.05.

#### Rip current

Prior experience with rip currents moderated the effect of actual water speed on perceived water speed (*b* = −3.18, *t*_73_  = −3.35, *p* = .001, *r*_p_  = −.37); the relationship for people with no rip current experience was significantly more positive than it was for people with rip current experience (Table 5). Controlling for rip current experience, the direct and indirect effects of water speed accounted for 56% and 44% of the total effect, respectively. For people with no rip current experience, mediation was stronger (direct and indirect effects accounted for 56% and 44% of the total effect), than it was for people with rip current experience (direct and indirect effects accounted for 59% and 41% of the total effect).

#### Number of storms

Prior experience with storms moderated the effect of actual water speed on perceived water speed (*b* = −0.38, *t*_73_  = −2.14, *p* = .032, *r*_p_  = −.24); the relationship for people with no storm experience was significantly more positive than it was for people who had experienced 10 storms or more (Table 6). At the mean number of storms experienced (5 storms), the direct and indirect effects of water speed accounted for 56% and 44% of the total effect, respectively. For people with no storm experience, mediation was slightly weaker (direct and indirect effects accounted for 58% and 42% of the total effect), than it was for people who had experienced 10 storms or more (direct and indirect effects accounted for 59% and 41% of the total effect).

## Discussion

Consistent with our first prediction, most people overestimated moving water speed, and departed even further from accuracy as either actual water speed increased, or when they were immersed in deeper water. These trends were also true for perceptions of personal risk. Supporting our second prediction, the direct relationship between actual water speed and risk perceptions was partially mediated by water speed perceptions; and, showing moderated mediation, this direct relationship was stronger for people with more prior storm or rip current experience. Together, these findings highlight not only people's inability to gauge water speed accurately (especially at higher speeds), but also the importance of individual differences in prior experience in shaping the accuracy of people's perceptions of moving water.

Despite these advances, the present research had limitations. First, we did not attempt to replicate turbulence beyond incorporating turning vanes in the corner upstream of the test section to suppress large-scale motions and to improve uniformity. The low-cost controlled water flume used in this experiment could not be tuned to simulate the complex hydrodynamics that naturally occur in large-scale flooding events such as flash floods and storm surges. A controlled environment, however, is necessary for systematically manipulating water depth and speed in an experiment. Second, although 76 participants may be a modest sample size, it exceeds the sample sizes of prior studies on flood risk and human stability, some of which relied on a single person. Third, we did not account for individual differences in personality traits such as narcissism, impulsivity, and sensation seeking, which have been linked to risk behavior [18], [19]. Fourth, all ratings were subjective self-reports. Future studies should strive to measure risk-related stress in the physiological domain (heart rate, blood pressure, galvanic skin response). Fifth, our sample was mostly male (76%) and fairly young (*M*_age_  = 23.5 years). Thus, the generalizability of our findings to the broader population – especially older ones – should be done with caution. Future studies should consider using more diverse samples.

Regarding the possible public policy implications of these findings, we stress that our results are preliminary and further research must be done to better understand risk perceptions related not only to moving water, but also to extreme winds [11]. Nevertheless, some cautious speculation may be warranted. First, these results could lay the groundwork for future research that examines how best to communicate the dangers of moving water to the public. For example, instead of just reporting expected storm surges or flood stage crests in feet and inches, civil agencies could attempt to communicate the combination of moving water height and speed in human terms. For example, “A storm surge or flash flood of *X* magnitude is sufficient to knock down a *Y*-pound person (or car, or large truck, etc.).” Moreover, we hope this research will help remind readers that the flash floods and storm surges that accompany tropical storms and hurricanes are often their most costly and deadly effects.

Flash flooding kills nearly 100 Americans annually, and storm surges from hurricanes can kill thousands. If we can understand people's perception of moving water and identify factors that contribute to their risk assessment, then we might be able to better calibrate their perceptions to reality through education and exposure. The present study is the first to systematically examine human water speed perception and risk with a controlled experiment. Although further studies will be needed before this line of research can inform public policy on warning systems, the present study lays the key foundation for future studies, which could eventually help save lives.
